# The Mediating Effect of Self-Efficacy and Coping Strategy in Relation to Job Stress and Psychological Well-Being of Home-Visiting Care Workers for Elderly during the COVID-19 Pandemic

**DOI:** 10.3390/ijerph191912164

**Published:** 2022-09-26

**Authors:** Hee-Kyung Kim, Jeong-Hyo Seo, Cheol-Hee Park

**Affiliations:** 1 Department of Nursing, Kongju National University, Gongju 32588, Korea; 2 Department of Nursing, Graduate School, Kongju National University, Gongju 32588, Korea

**Keywords:** job stress, psychological well-being, self-efficacy, coping, care worker, COVID-19, mediating effect

## Abstract

The purpose of this study was to analyze the mediating effect of self-efficacy and coping strategy in the relationship between job stress and the psychological well-being of care workers. The subjects were 112 home-visiting care workers, and data were collected at four home-visiting nursing centers in a metropolitan city and a small and medium-sized city from July to August 2022. The collected data were analyzed by descriptive statistics, *t*-test, ANOVA, Pearson’s correlation co-efficient, multiple linear regression, and Sobel test. The mean score of psychological well-being was 3.33 ± 0.46 out of a possible 5. The subject’s psychological well-being was correlated with self-efficacy (r = 0.64, *p* < 0.001), problem-solving-focused coping (r = 0.58, *p* < 0.001), social-support-seeking coping (r = 0.34, *p* < 0.001), job stress (r = −0.31, *p* = 0.001), avoidance-focused coping (r = −0.37, *p* < 0.001). Self-efficacy (Z = −4.92, *p* < 0.001), problem-solving-focused coping (Z = −2.56, *p* = 0.010), and avoidance-focused coping (Z = −3.07, *p* = 0.002) had a mediating effect in the relationship between job stress and psychological well-being of the subjects during the COVID-19 pandemic. Based on these results, the psychological well-being nursing intervention program for home-visiting care workers need to include job stress, problem-solving-focused coping, and avoidance-focused coping.

## 1. Introduction

Predicting a surge in the elderly population and an increase in health costs, Korea has decided on long-term care for the elderly and chronically ill since the Long-Term Care Insurance Act was enacted on 1 July 2008. Health and medical personnel who provide care include doctors, nurses, social workers, and care workers, and most (89.5%) of them are care workers [1]. Care workers are professional caregivers who have received professional education and obtained certificates from the nation. Care workers provide physical and household support services to the elderly in elderly care and home care facilities for the elderly who are unable to perform their daily lives independently due to dementia and stroke [2]. Most of the long-term care institutions (77.3%) are home-based, and care workers visit their homes to provide services such as physical activity, housework, and daily life support to the elderly living in the community to maintain and improve their current level of function and improve their quality of life [3]. Furthermore, the worker communicates and interacts with local residents and restores the social activity function of the elderly [4].

Meanwhile, in South Korea, in total there were 16,929,564 COVID-19 confirmed cases, including 31,828 imported cases during this period [5]. Prior to the spread of the vaccine, the proportion of confirmed elderly people aged 60 or older in September 2022 was more than 18.6% [6]. Despite the continued promotion of vaccination, the deaths of the elderly and critically ill patients increased. After COVID-19, not only the public but also the elderly suffered various emotional difficulties such as fear, anxiety, and frustration due to quarantine action guidelines, as external activities such as public facilities use, social gatherings, and family and family gatherings were limited. In particular, the elderly living alone, the elderly in the late 80s or older, the elderly with diseases, and the elderly in vulnerable groups felt more difficult [7]. Moreover, due to the change in the daily life of the elderly due to COVID-19, anxiety and fear of infection increased, and movement, physical activity, and social exchange were limited. In the case of the elderly with mobility difficulties, it was also difficult to purchase masks. In particular, the closure of the Senior Welfare Center, the place where the elderly gathered, reduced the amount of exercise for the elderly, lack of nutrition, and increased the sense of isolation [8].

During the COVID-19 pandemic, the difficulty of home-visiting care workers has increased. COVID-19 negatively affected employment insecurity, disaster insecurity, and work-doubling of care workers. In particular, home-visiting care workers were cut off in the early stages of COVID-19, with more elderly people trying to reduce contact with outsiders, mask chaos and quarantine supplies were inadequate, and stress from work such as nursing and infection prevention was increased [9]. Especially, the stress of infectious diseases had a significant effect on the job stress of care workers (work-related, relationship-related) [3]. Job stress such as role ambiguity and role conflict of home-visiting care workers was found to increase turnover intention [10].

In a study [11] on healthcare workers (HCWs) responding to pandemics, including home-visiting caregivers, the rapid spread of infectious diseases such as SARS, MERS, and COVID-19 put a lot of pressure on health managers and reduced psychological well-being. Therefore, in the case of care workers, they have been thoroughly disinfected and cared for the subjects with fear and anxiety that their germs may cause infection in vulnerable elderly people or chronically ill people, but some caregivers were exposed to infection without knowing the exact route. In particular, care workers are suffering from threats to health rights such as the risk of infection and anxiety and stress by providing face-to-face service to elderly people with underlying diseases due to the nature of their work [3]. Additionally, home-visiting care workers experienced job stress and depression [12], and in response to job stress, psychological responses such as increased blood pressure, physiological responses of muscle tension, job dissatisfaction, and tension, and behavioral responses such as low job competency, performance, and turnover occurred [13,14]. This job stress affects the mental and physical health of an individual, resulting in exhaustion of nursing care workers and negatively affecting the organization. Therefore, it is necessary to actively manage the stress experienced by caregivers while performing their duties [15].

Moreover, in performing their duties, job stress is greatly felt when they are in an environment or condition that is difficult to adapt to, such as COVID-19 [16], and psychological well-being is lowered [17], so they should be supported to reduce job stress and have psychological well-being. A similar study of psychological well-being in nursing care workers showed that health care providers, elderly spouses who cared for the elderly in Hong Kong, experienced stress and depression, while high-tensioned caregivers showed more negative emotions than low tensioned caregivers [18]. In addition, it is said that job stress such as role conflict, role loss, and customer conflict negatively affected psychological well-being such as self-acceptance and positive interpersonal relationship among restaurant employees who provide services to customers [19]. Psychological well-being is a concept that indicates how well an individual is functioning [20]. This psychological well-being can be increased when the caregiver accepts himself as he is, maintains a positive relationship with others, and exerts control over the surrounding environment with the ability to regulate behavior. Therefore, in order to reduce job stress of nursing care workers and increase psychological well-being during the COVID-19 period, we intend to devise measures to increase behavioral control.

First of all, self-efficacy was considered as this variable. The higher the job stress of care workers, such as role conflict, role ambiguity, and role difference, the lower the self-efficacy [21]. There was partial mediating effect of self-efficacy in the relationship between job stress and turnover intention, so the turnover intention could be lowered [22]. In addition, confidence and self-efficacy in the area of self-regulation influenced psychological well-being [23]. Additionally, self-efficacy was mediating in the relationship between job stress and psychological health [24].

We also considered the concept of coping strategies. The job competence of home-visiting care workers was a factor influencing problem-solving and emotion-focused coping among coping strategies [25], and stress coping strategies had a mediating effect in the relationship between job stress and exhaustion of nursing care workers [26]. In addition, problem-solving-focused coping and avoidance-focused coping were the main influencing factors on the psychological well-being of female service workers [27], and problem-solving-focused coping and emotion-focused coping also affected the psychological well-being of office workers [28]. In addition, considering that the higher the social support of home-visiting caregivers, the higher the psychological well-being [29]. It is necessary to analyze the psychological well-being including coping strategies of home-visiting caregivers.

Therefore, this study aims to analyze the mediating effect of self-efficacy and coping strategies (problem-solving-focused coping, social support-seeking coping, and avoidance-focused coping) in the relationship between job stress and psychological well-being for home-visiting caregivers. The specific objectives are as follows. 1. Identify the degree of job stress, self-efficacy, coping strategy (problem-solving-focused coping, social support-seeking coping, avoidance-focused coping) and psychological well-being of the subject. 2. Identify the relationship between the subject’s job stress, self-efficacy, coping strategies (problem-solving-focused coping, social support-seeking coping, avoidance-focused coping) and psychological well-being. 3. Analyze mediating effect of self-efficacy and coping strategy (problem-solving-focused coping, social support-seeking coping, and avoidance-focused coping) in the relationship between job stress and phychological well-bing.

## 2. Materials and Methods

### 2.1. Participants

The subjects of the study were 112 nursing care workers in charge of visiting nursing homes belonging to nursing centers for the elderly located in a metropolitan city and a small and medium-sized city. They are nursing care workers who have worked for at least 6 months as adult men and women aged 18 or older who understood the purpose of the study and voluntarily expressed their intention to participate and agreed in writing. The method of calculating the number of subjects was based on a study [30] that tested the previous mediating effect, and was also calculated using G-power 3.1. program [31]. The number of samples required to maintain 5 predictors, effect size 0.15, significance level 0.05, and power 0.90 for regression analysis was 108, and 113 people were investigated in consideration of the dropout rate of 5%. 112 copies were used for the final analysis, except for one copy of the inappropriate response.

### 2.2. Procedures

Permission was obtained from the heads of four home-visiting nursing centers for data collection. The purpose of the study, security matters, and anonymity were explained to the care worker in charge of visiting care belonging to the center. Care workers completed the self-rating questionnaires on job stress, self-efficacy, coping strategy, and psychological well-being (see details below) after signing a written consent form. The time required to fill out the questionnaire was about 10–15 min. The Institutional Review Board of Kongju National University, Gongju, South Korea (IRB No. KNU_IRB_2022-63) approved the study which was performed in accordance with the seventh and current [32] edition of the Declaration of Helsinki. Data collection for this study was conducted from 9 July 2022 to 30 August 2022.

### 2.3. Measures

#### 2.3.1. Job Stress

To assess job-related stress, participants completed the Korean version [33] of the questionnaire on job satisfaction and burnout [34]. The questionnaire consists of 9 items, and the sub-area consists of 3 questions of role conflict, 3 questions of role ambiguity, and 3 questions of role excession. Each question is a Likert 5-point scale of 1 point “not at all” to 5 points “very yes”, and the higher the score, the higher the job stress. In the study of Kim [33], the reliability Cronbach’s α was role conflict and role ambiguity 0.85. and role excession 0.62. The overall reliability of Cronbach’s α was 0.84. In this study, role conflict and role ambiguity were 0.92 and role excession 0.81 and the overall reliability was 0.93.

#### 2.3.2. Self-Efficacy

To assess self-efficacy, participants completed the Korean version [35] of the Self-Efficacy Scale [36]. The questionnaire consists of 19 items. Each question is a Likert 5-point scale of 5 points from 1 point “not at all” to 5 points “very yes,” meaning that the higher the score, the higher the self-efficacy. In the study of Park [35], Cronbach’s α was 0.82 and in this study 0.91.

#### 2.3.3. Coping Strategies: Problem-Solving, Social Support-Seeking, Avoidance-Focused Coping

As for the coping strategy, we used a tool that Shin and Kim [37] tested validation. The questionnaire consists of 33 items, and according to the type of coping style, it consists of three sub-scales: problem-solving-focused coping, social support-seeking coping, and avoidance-focused coping. The subscale is 11 questions each. Originally, it was a three-point scale, but after reviewing the validity of the researchers, each question was revised to a five-point scale. Each question is a Likert 5-point scale of 5 points from 1 point of ‘not doing it at all’ to 5 points of ‘very much’. The higher the score of each coping style, the higher the degree of problem-solving-focused coping, the higher the degree of social support-seeking coping, and the higher the degree of avoidance-focused coping. In the study of Shin and Kim [37], the reliability of problem-solving-focused coping Cronbach’s α was 0.88, social support-seeking coping was 0.90, and avoidance-focused coping was 0.67. Problem-solving-focused coping in this study was 0.95, the social support pursuit coping was 0.88, and the avoidance-focused coping was 0.82.

#### 2.3.4. Psychological Well-Being

To assess psychological well-being, Participants completed a tool that PWBS [20] turned into a Korean version [38] and made for workers in welfare facilities [13]. With a total of 26 questions, the sub-area consists of 6 questions for self-acceptance, 5 questions for positive interpersonal relations, 4 questions for autonomy, 3 questions for control over the environment, 4 questions for life purpose, and 4 questions for personal growth. Each question is a Likert 5-point scale of 1 point “not at all” to 5 points “very yes,” meaning that the higher the score, the higher the psychological well-being. In the study of Kim [13], the self-acceptance reliability Cronbach’s α in the sub-area of psychological well-being was 0.78, positive interpersonal relationship 0.77, autonomy 0.36, control over the environment 0.64, purpose of life 0.78, personal growth 0.70, and overall reliability Cronbach’s α value 0.84. In this study, the self-acceptance reliability Cronbach’s α was 0.85, positive interpersonal 0.72, autonomy 0.52, control over the environment 0.86, purpose of life 0.75, personal growth 0.74, and overall reliability Cronbach’s α value was 0.88.

### 2.4. Statistical Analyses

The collected data were analyzed using the SPSS^®^ statistics for windows 25.0 (IBM Corporation, Armonk, NY, USA).

First, the inspection with a series of Kolmogorov–Smirnov tests, skewness and kurtosis showed that outcome variables were normally distributed.

With descriptive statistics such as frequency, percentage, mean and standard deviation, the general characteristics of the subjects and the degree of variables were calculated.

With *t*-test and ANOVA, we compared with psychological well-being according to the general characteristics of the subjects.

With Pearson’s correlation co-efficients, the relationship between job stress, self-efficacy, problem-solving-centered coping, social support-seeking coping, avoidance-focused coping and psychological well-being of subjects were calcurated.

With multiple linear regression, the mediating effect of self-efficacy and coping strategy (problem-solving-focused coping, social support-seeking coping, and avoidance-focused coping) were analyzed. In order to test the statistical significance of the mediating effect, Sobel test was used.

### 2.5. Ethical Principles

In order to collect data for this study, a research plan was submitted to the Institute Review Board of K University and deliberation exemption was received (KNU_IRB_2022-63). This researcher informed the subjects that they were free to not participate in this study, that there was no disadvantage even if they did not participate, and that they could stop participating at any time if they did not want to. Data collected during the study were stored in a personal locker with a lock that can only be used by researchers, and the data will be deleted three years after the end of the study.

## 3. Results

### 3.1. General Information of Participants

Table 1 shows participants’ sociodemographic and working-related information. The age of the participants is 34–80 years, and the average age was 61.35 ± 7.20 years, and 63.4% (71 people) were over 60. Among them, women was 95.5 percent (107 people). In terms of marital status, 96.4% (108 people) were married, 73.2% (81 people) said they had religion, and 84.8% (95 people) were under high school graduation. The average working experience was 5.12 ± 3.62 years, and 50.9% (57 people) were less than 5 years. 68.8% (77 people) of the subjects answered that they had only a nursing care worker certificate, and 91.2% (101 people) said they had never received education on psychological well-being. Monthly income was 64.3% (72 people) with less than 1 million won, and 97.3% (109 people) were eligible to perform the duties of visiting care only.

### 3.2. Differences in Psychological Well-Being According to the General Information of Participants

Table 1 shows the difference in psychological well-being according to the sociodemographic and working-related information. There was a difference in psychological well-being according to the degree of education of the subject (t = −3.05, *p* = 0.003). In other words, subjects with educational background above college graduation showed a higher degree of psychological well-being at a statistically significant level than middle and high school graduates.

### 3.3. Degree of Job Stress, Self-Efficacy, Problem-Solving-Focused Coping, Social Support-Seeking Coping, Avoidance-Focused Coping and Psychological Well-Being of Participants and Relation of Variables

In the Kolmogorov–Smirnov normality test of all variables, the *p* value was greater than 0.05, the skewness was −0.60 to 0.31, and the absolute value was less than 2, and the kurtosis was −0.30 to 1.41, satisfying the univariate normality assumption of the sample.

Table 2 shows the degree of the psychological well-being and related variables of participants. The average score of the subject’s job stress was 2.54 ± 0.94 out of 5. The average score of self-efficacy was 3.84 ± 0.51 out of 5. Among the coping strategies, the average score of problem-solving-focused coping was 3.77 ± 0.62 out of 5, the average score of social support-seeking coping was 3.56 ± 0.57 out of 5, and the average score of avoidance-focused coping was 2.29 ± 0.52 out of 5. The average score of psychological well-being was 3.33 ± 0.46 out of 5.

### 3.4. Relations of Job Stress, Self-Efficacy, Problem-Solving-Focused Coping, Social Support-Seeking Coping, Avoidance-Focused Coping and Psychological Well-Being of Participants

Table 2 shows the relation between the participants’ psychological well-being and variables. The participants’ psychological well-being had a statistically significant positive correlation between self-efficacy (r = 0.64, *p* < 0.001), problem-solving-focused coping (r = 0.58, *p* < 0.001), and social support-seeking coping (r = 0.34, *p* < 0.001). There was a statistically significant negative correlation with job stress (r = −0.31, *p* = 0.001), avoidance-focused coping (r = −0.37, *p* < 0.001). In other words, it can be seen that the higher the participants’ self-efficacy, the higher the psychological well-being when there is stress, the more problem-solving-focused coping, and the more social support-seeking coping are used. On the other hand, it was found that the higher the job stress, the lower the psychological well-being was when there was stress and the more avoidance-focused coping were used.

### 3.5. Mediating Effect of Self-Efficacy in the Relation between Job Stress and Psychological Well-Being of Participants

Prior to the mediating effect test, the assumption of regression analysis was tested. As a result of examining the residual plot for the equivariance test, equivariance was confirmed, and the Durbin-Watson value for verifying the independence of the residuals was 1.605, close to 2, satisfying the independence assumption. As a result of examining the P-P chart to confirm independence to verify the normality of the error term, the normal distribution was shown. In addition, in the evaluation of multicollinearity between independent variables, the tolerance was 0.44–0.79, and the variance expansion factor (VIF) of variables was 1.26–2.30, which was less than 10, so the basic assumptions of the equivalence and normal distribution of the residuals were satisfied.

Table 3 provides the mediating effect of self-efficacy in the relationship between job stress and psychological well-being of the participants.

**Figure 1:**
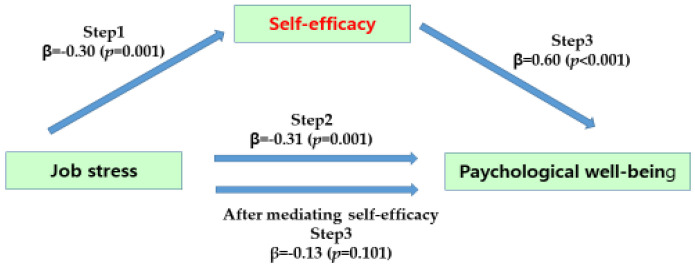
Mediating effect of self-efficacy of participants.

In step 1, job stress, an independent variable, had a statistically significant effect on self-efficacy, which is a parameter (β = −0.30, *p* = 0.001), and in step 2, job stress, an independent variable, had a statistically significant effect on psychological well-being, which is a dependent variable (β = −0.31, *p* = 0.001). In step 3, job stress, an independent variable, and self-efficacy, a parameter, were simultaneously put into the regression model to predict psychological well-being, and as a result, self-efficacy had a significant effect on psychological well-being (β = −0.60, *p* < 0.001). The regression coefficient of job stress on psychological well-being decreased from −0.31 to −0.13 but was not statistically significant (β = −0.13, *p* = 0.101); thus, self-efficacy was found to have a complete mediating effect in the relationship between job stress and psychological well-being. As a result of testing the significance of the mediating effect in the Sobel test result, it was statistically significant (Z = −4.92, *p* < 0.001) (Figure 1).

### 3.6. Mediating Effect of Problem-Solving-Focused Coping, Social Support-Seeking Coping, Avoidance-Focused Coping in the Relation between Job Stress and Psychological Well-Being of Participants

Table 4 provides the mediating effect of problem-solving-focused coping in the relationship between job stress and psychological well-being of the subjects.

**Figure 2:**
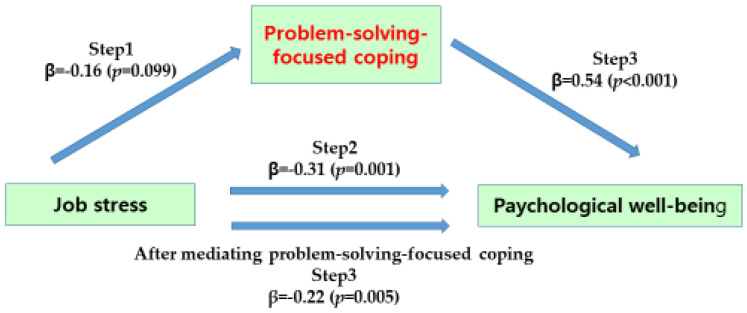
Mediating effect of problem-solving-focused coping of participants.

In step 1, there was a statistically significant effect on the problem-solving-centered coping with the independent variable as a parameter (β = −0.16; *p* = 0.099), and in step 2, the independent variable had a statistically significant effect on the psychological well-being with the dependent variable (β = −0.31, *p* = 0.001). In step 3, as a result of predicting psychological well-being by simultaneously putting independent variable job stress and parameter problem-solving-centered coping into the regression model, problem-solving-centered coping had a significant effect on psychological well-being (β = 0.54, *p* < 0.001). The regression coefficient of job stress on psychological well-being decreased from −0.31 to −0.22 and was statistically significant (β = −0.22, *p* = 0.005). Therefore, in the relationship between job stress and psychological well-being, problem-solving-centered coping was found to have a partial mediating effect, and as a result of testing the significance of the mediating effect in the Sobel test results, it was statistically significant (Z = −2.56, *p* = 0.010) (Figure 2).

Table 5 provides the mediating effect of social support-seeking coping in the relationship between job stress and psychological well-being of the participants.

In step 1, job stress, an independent variable, was not statistically significant in coping with social support, which is a parameter. In step 2, job stress, an independent variable, had a statistically significant effect on psychological well-being, a dependent variable (β = −0.31, *p* = 0.001). The effect between the independent variable and the parameter in step 1 is not statistically significant, so the statistical treatment in step 3 is meaningless.

Table 6 provides the mediating effect of avoidance-focused coping in the relationship between job stress and psychological well-being of the participants.

**Figure 3:**
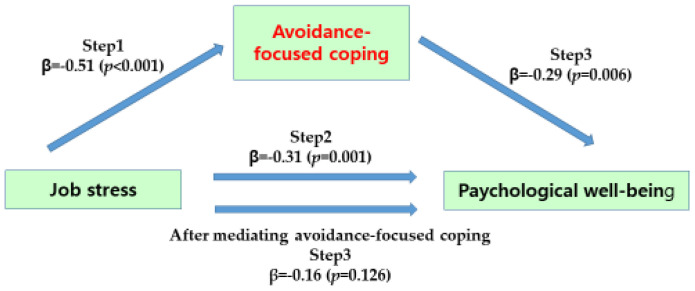
Mediating effect of avoidance-focused coping of participants.

In step 1, job stress, an independent variable, had a statistically significant effect on coping with avoidance-centeredness as a parameter (β = 0.51, *p* < 0.001), and in step 2, job stress, an independent variable, had a statistically significant effect on psychological well-being as a dependent variable (β = −0.31, *p* = 0.001). In step 3, as a result of predicting psychological well-being by simultaneously putting independent variable job stress and parameter avoidance-focused coping into the regression model, avoidance-focused coping had a significant effect on psychological well-being (β = −0.29, *p* = 0.006). The regression coefficient of job stress on psychological well-being decreased from −0.31 to −0.16 but was not statistically significant (β = −0.16, *p* = 0.126). Therefore, in the relationship between job stress and psychological well-being, avoidance-focused coping was found to have a complete mediating effect, and as a result of testing the significance of the mediating effect in the Sobel test results, it was statistically significant (Z = −3.07, *p* = 0.002) (Figure 3).

## 4. Discussion

This study attempted to understand the relationship between job stress, self-efficacy, stress coping strategy and psychological well-being of home-visiting care workers during the COVID-19 pandemic, and to identify the mediating effect of self-efficacy, problem-solving-focused coping, social support-seeking coping, and avoidance-focused coping in relation to job stress and psychological well-being.

In this study, the job stress of nursing care workers was 2.54 out of 5. As a result of a study by Lee, Ku [39] on female nursing care workers at home welfare centers during COVID-19, it was 2.86 points when converting job stress to 5 points, and 2.71 points in Yoon [40], and the job stress of female service workers was 2.63 [27] which was similar to this study. Meanwhile, the job stress scores during COVID-19 were somewhat higher than 2.3 points of Kim [33] for elderly care workers in the pre-COVID-19 period. During the COVID-19 pandemic, the job stress of home-visiting care workers is stress on care such as fear of infection, social distancing, and care for vulnerable subjects [3], so measures are required to reduce it.

The degree of psychological well-being of nursing care workers was 3.33 points. On the other hand, women in their 20 s and 60 s before COVID-19 scored 3.91 points [38], higher than the results of this study, which also suggests that the degree of psychological well-being can be lowered due to psychological instability and fear of infectious diseases. The positive perception of stress events is helpful for psychological well-being, and when psychological well-being is high, stress is perceived low [17,27]. So measures should be devised to lower job stress and increase psychological well-being.

In the relationship between job stress and psychological well-being of home-visiting nursing care workers, self-efficacy had a complete mediating effect. This was consistent with the result [24] of reporting that self-efficacy was a mediating effect in the relationship between job stress and mental health of nursing care workers visiting nursing hospitals and home visits, and self-efficacy of adults was found to be a major influence [25] on psychological well-being. A care workers must be confident that he or she can perform his or her role in various situations [25] because he or she must provide services immediately. Therefore, self-efficacy can complete the role and increase satisfaction by exercising job competency with psychological well-being even in job stress situations, so a strategy to increase self-efficacy is required. Bandura [41] cited achievement, surrogate experience, verbal persuasion, and physiological status as sources of information that affect expectations for self-efficacy. Performance achievement is based on mastery experiences, so successful performance should be achieved through these experiences. In addition, expectations for self-efficacy can be increased by seeing others perform successfully through proxy experience. Verbal persuasion such as advice and advice from others and creating a physiological state rather than a high stimulation state can also increase self-efficacy. Therefore, it is necessary to develop and apply a customized self-efficacy promotion program considering these factors to home-visiting nursing care workers.

As for stress coping strategies, Lazarus and Folkman [42] stated that stress coping strategies are more significant than stress itself and have an important effect on adaptation. Active coping includes solving problems while overcoming setbacks or obstacles, and passive coping includes emotional mitigation, wishful thinking, and problem avoidance as an effort to avoid or defend problems [25]. Stress coping ability has a positive effect on psychological well-being, and among the sub-areas of stress coping ability, active coping such as problem-solving and social support-seeking coping has higher psychological well-being, and passive coping such as avoidance-focused coping has lower psychological well-being [23].

As a result of this study, it was found that problem-solving-focused coping had a partial mediating effect in the relationship between job stress and psychological well-being of nursing care workers. Among health managers during the COVID-19 pandemic, nurses who take care of patients face-to-face for the longest time were the most stressful, but they also had high coping ability, so using appropriate coping strategies for stress [43] is an important factor in stress management. In addition, during the COVID-19 pandemic, health care workers’ coping strategies had a positive effect on psychological and mental health [44]. Problem-solving-focused coping was the factor that had the greatest influence on the psychological well-being of female service workers during the COVID-19 period [27]. Job stress and problem-solving-focused coping of office workers were found to be major influencing factors on psychological well-being [28], and this study was supported in that approaching problem-solving methods among stress coping strategies can give psychological well-being. Problem-solving-centered coping is a positive way of approaching an individual actively with the will to solve a problem when he encounters difficulties [25]. It is said that caregivers who care for vulnerable subjects such as people with developmental disabilities play a protective role in reducing burnout when using problem-solving-focused coping [45]. Therefore, in a situation where many people have health problems such as stress, anxiety, and depression due to the long-term extension of the infectious disease period, problem-solving-focused coping can help home-visiting caregivers to gain psychological well-being. Managers should support home-visiting care workers to build coping skills. Considering that mindfulness training has decreased and problem-oriented response has increased in longitudinal studies of college students [46], mindfulness training can help improve nursing care workers’ coping skills and improve psychological well-being.

Next, in the relationship between job stress and psychological well-being of care workers, avoidance-focused coping was found to have a complete mediating effect. Because avoidance-focused coping was a major factor in the psychological well-being of female service workers during the COVID-19 period [27], and avoidance-focused coping had a mediating effect in the relationship between stress and well-being of nursing college students [47], some studies are consistent with the results of this study. However, Avoidance-focused coping is a strategy that avoids or does not experience the idea of stress [48]. It may help to temporarily lower stress, but in the long run, it is not suitable for improving psychological well-being and rather can lead to psychological difficulties [47]. In this study, avoidance-focused coping should not be used because they have an inverse correlation with psychological well-being, resulting in negative results in psychological well-being even if they have a complete mediating effect. In the study of Navill and Havercamp [45], avoidance-focused coping was also found to be a risk factor. Therefore, since the avoidance-focused coping shown in the results of this study can have negative results, education and training are needed for home-visiting care workers to use problem-solving-focused coping rather than avoidance-focused response as a coping strategy.

Meanwhile, in this study, it was found that social support-seeking coping had no mediating effect on the job stress and psychological well-being of home-visiting care workers. The social support and psychological well-being of home care caregivers had a high correlation and affected job satisfaction. Home-visiting nursing care workers need resources to restore physical and psychological health, and social support from colleagues and families can be said to be of great help in restoring psychological well-being [29]. Therefore, continuous research on social support response is needed for home-visiting nursing care workers.

This study targets home-visiting nursing care workers in some regions, so it is necessary to pay attention when expanding interpretation to all nursing care workers.

## 5. Conclusions

During the COVID-19 pandemic. In the relationship between job stress and psychological well-being of home-visiting nursing care workers, self-efficacy had a complete mediating effect. Among the coping strategies, problem-solving-focused coping had a partial mediating effect, and avoidance-focused coping had a complete mediating effect. Overall, it is necessary to consider self-efficacy, self-efficacy, problem-solving-focused coping, and short-term avoidance-focused coping when devising methods to lower job stress and increase psychological well-being of home-visiting care workers.

## Figures and Tables

**Table 1:** Sociodemographic and working-related information of the participants and differences in psychological well-being according to general information.

(*N* = 112)							
Variables	Classification	*n*	%	Psychological Well-Being			
				Mean	SD	t/F	*p*-Value Scheffe Test
Age	Under 50	4	3.6	3.53	0.32	0.76	0.472
	50–59	37	33.0	3.37	0.44		
	Over 60	71	63.4	3.29	0.47		
Gender	Female	107	95.5	3.33	0.45	−0.02	0.983
	Male	5	4.5	3.33	0.58		
Marital status	Married	108	96.4	3.34	0.46	1.64	0.104
	Unmarried, Divorce, etc.	4	3.6	2.96	0.12		
Religion	Yes	81	73.2	3.32	0.45	−0.29	0.773
	No	30	26.8	3.34	0.49		
Education	Under high school	95	84.8	3.27	0.42	−3.05	0.003
	college graduation or higher	17	15.2	3.63	0.54		
Career experience	Below 5 years	57	50.9	3.32	0.48	−0.79	0.937
	Over 5 years	55	49.1	3.33	0.44		
Certi except for nursing care	No	77	68.8	3.31	0.48	−0.45	0.656
	Yes	34	31.2	3.35	0.40		
Education on phychological well-being	No	101	91.2	3.33	0.46	−0.16	0.987
	Yes	11	9.8	3.33	0.46		
Monthly income	Below 1 million won	72	64.3	3.30	0.48	−0.77	0.444
	More than 1 million won	40	35.7	3.37	0.41		
Working type	Visiting care	109	97.3	3.33	0.46	−0.22	0.824
	Visiting care and bathing	3	2.7	3.38	0.43		

**Table 2:** Degree of job stress, self-efficacy, problem-solving-focused coping, social support-seeking coping, avoidance-focused coping and psychological well-being of participants and relations of the variables.

(*N* = 112)						
Variables	Job Stress r (*p*)	Self-Efficacy r (*p*)	Problem-Solving -Focused Coping r (*p*)	Social Support -Seeking Coping r (*p*)	Avoidance-Focused Coping r (*p*)	Psychological Well-Being r (*p*)
Job stress	1					
Self-efficacy	−0.30 (0.001)	1				
Problem-solving -focused coping	−0.16 (0.099)	0.68 (<0.001)	1			
Social support -seeking coping	−0.13 (0.177)	0.19 (0.051)	0.41 (<0.001)	1		
Avoidance-focused Coping	0.51 (<0.001)	−0.40 (<0.001)	−0.16 (0.103)	−0.15 (0.105)	1	
Psychological well-being	−0.31 (0.001)	0.64 (<0.001)	0.58 (<0.001)	0.34 (<0.001)	−0.37 (<0.001)	1
Mean	2.54	3.84	3.77	3.56	2.29	3.33
SD	0.94	0.51	0.62	0.57	0.52	0.46

**Table 3:** Mediating effects of self-efficacy in the relation between job stress and psychological well-being in participants.

Variables	B	SE	β	T(*p*)	R^2^	Adj. R^2^	F(*p*)
Step1: Job stress → Self-efficacy	−0.16	0.05	−0.30	−3.29 (0.001)	0.090	0.081	10.82 (0.001)
Step2: Job stress → Psychological well-being	−0.15	0.04	−0.31	−3.37 (0.001)	0.094	0.086	11.38 (0.001)
Step3: Job stress, Self-efficacy → Psychological well-being	−0.06 0.54	0.04 0.07	−0.13 0.60	−1.66 (0.101) 7.90 (<0.001)	0.424	0.413	40.04 (<0.001)

**Table 4:** Mediating effects of problem-solving focused coping in the relation between job stress and psychological well-being in participants.

Variables	B	SE	β	T(*p*)	R^2^	Adj. R^2^	F(*p*)
Step1: Job stress → Problem-solving-focused coping	−0.10	0.06	−0.16	−1.66 (0.099)	0.025	0.016	2.77 (0.099)
Step2: Job stress → Psychological well-being	−0.15	0.04	−0.31	−3.37 (0.001)	0.094	0.086	11.38 (0.001)
Step3: Job stress, Problem-solving-focused coping → Psychological well-being	−0.11 0.40	0.04 0.06	−0.22 0.54	2.90 (0.005) 7.09 (<0.001)	0.380	0.368	33.36 (<0.001)

**Table 5:** Mediating effects of social support-seeking coping in the relation between job stress and psychological well-being in participants.

Variables	B	SE	β	T(*p*)	R^2^	Adj. R^2^	F(*p*)
Step1: Job stress → Social Support-seeking coping	−0.08	0.06	−0.13	−1.36 (0.177)	0.016	0.008	1.843 (0.177)
Step2: Job stress → Psychological well-being	−0.15	0.04	−0.31	−3.37 (0.001)	0.094	0.086	11.379 (0.001)
Step3: Job stress, Social support-seeking coping → Psychological well-being	−0.13 0.24	0.04 0.07	−0.27 0.30	−3.06 (0.003) 3.47 (0.001)	0.184	0.169	12.267 (<0.001)

**Table 6:** Mediating effects of avoidance-focusing coping in the relation between job stress and psychological well-being in participants.

Variables	B	SE	β	T(*p*)	R^2^	Adj. R^2^	F(*p*)
Step1: Job stress → Avoidance-focusing coping	0.28	0.05	0.51	6.29 (<0.001)	0.264	0.258	39.531 (<0.001)
Step2: Job stress → Psychological well-being	−0.15	0.04	−0.31	−3.37 (0.001)	0.094	0.086	11.379 (0.001)
Step3: Job stress, Avoidance-focusing coping → Psychological well-being	−0.08 −0.25	0.05 0.09	−0.16 −0.29	−1.54 (0.126) −2.80 (0.006)	0.155	0.139	9.975 (<0.001)

## Data Availability

The data underlying this article will be shared upon reasonable request from the corresponding author.

## References

[1] National Health Insurance (2021). 2020 Statistical Yearbook of Long-Term Care Insurance for the Elderly.

[2] Ministry of Health and Welfare Guidelines for Training Care Workers in 2022. Publishing Registration no. 11-1352000-002419-10. Ministry of Health and Welfare, 2022. http://www.mohw.go.kr/react/jb/sjb030301vw.jsp?PAR_MENU_ID=03&MENU_ID=0320&CONT_SEQ=370618.

[3] Lee J.W. (2021). Influence of Infectious Disease Stress on Job Stress of Personal Care Assistants. Master’s Thesis.

[4] Lim K.K. (2011). Job Satisfaction of the Paid Care Givers Working in Facilities and Homes for the Elderly’s Welfare. Master’s Thesis.

[5] Korea Disease Control and Prevention Agency (2022). [COVID-19 Special Report] Outbreak report of COVID-19 during designation of class 1 infectious disease in the Republic of Korea (January 20, 2020–April 24, 2022). Wkly. Health Dis..

[6] Korea Disease Control and Prevention Agency (2022). COVID-19 Domestic Occurrence Status. http://ncov.mohw.go.kr/bdBoardList_Real.do?brdId=1&brdGubun=11&ncvContSeq=&contSeq=&board_id=&gubun=.

[7] Namkung E.H. (2021). Social and Economic Experiences and Health Changes for Older Persons during the COVID-19 Pandemic. Health Welf. Policy Forum.

[8] Shin H.R., Yoon T.Y., Kim S.K., Kim Y.S. (2020). An exploratory study on changes in daily life of the elderly amid COVID-19—Focusing on technology use and restrictions on participation in elderly welfare centers. Korean J. Gerontol. Soc. Welf..

[9] Kweon G.D. Care Workers Have Increased Their Work Due to COVID-19, but Treatment Is Poor… 9.2% ↑ of Sexual Violence from 8 Years Ago. Hankyoreh, 24 November 2021.

[10] Lee J.Y. (2018). Effects of domiciliary care center visiting care provider’s job stress on turnover intention—Exhaustion as the mediation effect. J. Soc. Welf. Manag..

[11] Schneider J., Talamonti D., Gibson B., Forshaw M. (2022). Factors mediating the psychological well-being of healthcare workers responding to global pandemics: A systematic review. J. Health Psychol..

[12] Jeon G.S., You S.J., Kim M.G., Kim Y.M. (2017). Correlates of depressive symptoms and stress among Korean women care-workers for older adults dwelling in community. Korean J. Occup. Health Nurs..

[13] Kim K.H. (2012). The Mediating e Effect of Job Stress on the Relationship between Job Characteristics and Psychological Well-Being of Workers at Senior Welfare Facilities. Master’s Thesis.

[14] Park C.H. (2021). Effects of Fatigue, Job Stress, and Empathic Ability on the Job Competency of Home-Visit Caregivers. Master’s Thesis.

[15] Lee Y.H., Lim W.K. (2011). Effects of the long-term caregivers’ job stress on psychological burnout and organizational effectiveness and the moderating role of social support. J. Korea Contents Assoc..

[16] Jondhale A., Anap D. (2012). Job stress among the nursing staff working in rural health care set up. Int. J. Nurs. Educ..

[17] Huh J.A., Kim J.M. (2017). The relation between self-encouragement, perceived stress and psychological well-being: The moderated mediating effect of support-seeking emotion regulation style. Stress.

[18] Chow E.Q., Ho H.C. (2015). Caregiver strain, age, and psychological well-being of older spousal caregivers in Hong Kong. J. Soc. Work.

[19] Lee C.M., Yoon H.H. (2019). A study on the relationship between employee’s job stress, psychological well-being and, job satisfac-tion, turnover intention in foodservice employees: Moderating effect of resilience. J. Foodserv. Manag..

[20] Ryff C.D. (1989). Happiness is everything, or is it? Explorations on the meaning of psychological well-being. J. Personal. Soc. Psychol..

[21] Seong J.A. (2020). The effect of self-efficacy and job stress on quality of service of care givers. J. Converg. Inf. Technol..

[22] Baek S.Y. (2019). The effects of job stress on the self-efficacy and change job nursing care institution. J. Humanit. Soc. Sci..

[23] Kim K.S. (2011). The Influence of Self-Efficacy, Stress Management, and Psychological Well-Being on Subjective Well-Being. Doctoral Dissertation.

[24] Lee M.L. (2012). Effects of Self-Efficacy in the Relationships between Job Stress and Mental Fitness of Care Provider. J. Korea Contents Assoc..

[25] Kim A.R. (2019). Impacts of Care Worker’s Self-Efficacy and Stress-Coping Ability on Job Competency: Focused on Female Care Workers Providing Home Care Services in the C. Region. Master’s Thesis.

[26] Moon H. (2010). The mediator effect of coping strategies in the relationship between care givers’ job stress and burnout. Korean J. Community Living Sci..

[27] Kim H.K. (2022). In the COVID-19 era, effects of job stress, coping strategies, meaning in life and resilience on psychological well-being of women workers in the service sector. Int. J. Environ. Res. Public Health.

[28] Jo S., Ko D.W., Lee J.Y. (2018). Relationship among job stress, stress coping strategy method and psychological well-being of office workers. Bus. Case Rev..

[29] Lee S.M. (2019). The Effects of Social Support on the psychological Well-being of In-Home Care Workers: With a focus on the medi-ating effects of job satisfaction. Korean J. Care Manag..

[30] Shin S.H. (2018). Mediating effects of self-reassuring and self-attacking on relationship between mental health and psychological well-being among college students. J. Korea Contents Assoc..

[31] Faul F., Erdfelder E., Buchner A., Lang A.G. (2009). Statistical power analyses using G*Power 3.1: Tests for correlation and regression analyses. Behav. Res. Methods.

[32] World Medical Association (2013). World medical association declaration of Helsinki. Ethical principles for medical research involving human subjects. JAMA.

[33] Kim M.S. (2017). A Study on the Influences of Emotional Work and Job Stress on Burnout and Customer Orientation of Elderly Care Workers. Doctoral Dissertation.

[34] Jayaratne S., Chess W.A., Farber B.A. (1983). Job satisfaction and burnout in social work. Stress and Burnout in the Human Service Professions.

[35] Park J.I. (2008). The Moderating Effects of Spiritual Well-Being on Self-Efficacy and Job Satisfaction of the Social Workers. Doctoral Dissertation.

[36] Sherer M., Maddux J.E., Meracndante B., Prentice-Dunn S., Jacops B., Rogers R.W. (1982). The self-efficacy scale: Construction and validation. Psychol. Rep..

[37] Shin H.J., Kim C.D. (2002). A validation study of coping strategy indicator (CSI). Korean J. Couns. Psychother..

[38] Kim M.S., Kim H.W., Cha K.H. (2001). Analysis on the construct of psychological well-being (PWS) of Korean male and woman adults. Korean J. Soc. Personal. Psychol..

[39] Lee E.A., Ku H.S. (2020). The study on the effects of job stress of female caregivers in home-based elderly welfare center on turnover intention: Self-elasticity as a controlling variable. J. Ind. Converg..

[40] Yoon Y.S. (2019). Burnout among Care Helpers in Home Service Centers: Focusing on the Impact of Job Stress, Calling, and Supervision. Master’s Thesis.

[41] Bandura A. (1977). Self-efficacy: Toward a unifying theory of behavioral change. Psychol. Rev..

[42] Lazarus R., Folkman S. (1984). Stress, Appraisal, and Coping.

[43] Croghan I.T., Chesak S.S., Adusumalli J., Fischer K.M., Beck E.W., Patel S.R., Ghosh K., Schroeder D.R., Bhagra A. (2021). Stress, resilience, and coping of healthcare workers during the COVID-19 pandemic. J. Prim. Care Community Health.

[44] Labrague L.J. (2021). Psychological resilience, coping behaviours and social support among health care workers during the COVID-19 pandemic: A systematic review of quantitative studies. J. Nurs. Manag..

[45] Nevill R.E., Havercamp S.M. (2019). Effects of mindfulness, coping styles and resilience on job retention and burnout in caregivers supporting aggressive adults with developmental disabilities. J. Intellect. Disabil. Res..

[46] De Vibe M., Solhaug I., Rosenvinge J.H., Tyssen R., Hanley A., Garland E. (2018). Six-year positive effects of a mindfulness-based intervention on mindfulness, coping and well-being in medical and psychology students; Results from a randomized controlled trial. PLoS ONE..

[47] Lee E.S. (2017). Impact of life stress on depression, subjective well-being and psychological well-being in nursing students: Mediation effects of coping. J. Korea Acad.-Ind. Coop. Soc..

[48] Amirkhan J.H. (1990). A factor analytically derived measure of coping: The coping strategy indicator. J. Personal. Soc. Psychol..

